# Delayed degradation of chlorophylls and photosynthetic proteins in *Arabidopsis* autophagy mutants during stress-induced leaf yellowing

**DOI:** 10.1093/jxb/eru008

**Published:** 2014-02-08

**Authors:** Yasuhito Sakuraba, Sang-Hwa Lee, Ye-Sol Kim, Ohkmae K. Park, Stefan Hörtensteiner, Nam-Chon Paek

**Affiliations:** ^1^Department of Plant Science, Plant Genomics and Breeding Institute, and Research Institute for Agriculture and Life Sciences, Seoul National University, Seoul 151-921, Korea; ^2^Department of Life Sciences, Korea University, Seoul 136-701, Korea; ^3^Institute of Plant Biology, University of Zurich, CH-8008 Zurich, Switzerland

**Keywords:** Abiotic stress, *Arabidopsis thaliana*, autophagy, *atg5*, chlorophyll degradation, leaf senescence, stay-green.

## Abstract

Plant autophagy, one of the essential proteolysis systems, balances proteome and nutrient levels in cells of the whole plant. Autophagy has been studied by analysing *Arabidopsis thaliana* autophagy-defective *atg* mutants, but the relationship between autophagy and chlorophyll (Chl) breakdown during stress-induced leaf yellowing remains unclear. During natural senescence or under abiotic-stress conditions, extensive cell death and early yellowing occurs in the leaves of *atg* mutants. A new finding is revealed that *atg5* and *atg7* mutants exhibit a functional stay-green phenotype under mild abiotic-stress conditions, but leaf yellowing proceeds normally in wild-type leaves under these conditions. Under mild salt stress, *atg5* leaves retained high levels of Chls and all photosystem proteins and maintained a normal chloroplast structure. Furthermore, a double mutant of *atg5* and non-functional stay-green *nonyellowing1-1* (*atg5 nye1-1*) showed a much stronger stay-green phenotype than either single mutant. Taking these results together, it is proposed that autophagy functions in the non-selective catabolism of Chls and photosynthetic proteins during stress-induced leaf yellowing, in addition to the selective degradation of Chl–apoprotein complexes in the chloroplasts through the senescence-induced STAY-GREEN1/NYE1 and Chl catabolic enzymes.

## Summary

Under mild abiotic-stress conditions, *Arabidopsis atg* mutants showed a functional stay-green phenotype which is probably caused by the lack of chloroplastic autophagy and the retrograde regulation of senescence-associated gene expression.

## Introduction

Senescence marks the final stage of leaf development in plants. In the early phase of leaf senescence, developmental and environmental cues signal the plant cells to activate transcription factors (TFs) that modulate the expression of senescence-associated genes (SAGs) (Guo and Ecker, 2004; Balazadeh *et al.*, 2008). The products of these SAGs conduct the highly ordered breakdown of intracellular organelles, including the degradation of proteins and macromolecules to remobilize leaf nutrients into other developing organs such as new leaves or seeds, or into storage organs (Lim *et al.*, 2007; Robinson *et al.*, 2012).

Autophagy, a highly conserved process in eukaryotes, functions as one of the major pathways for the massive degradation of intracellular proteins during leaf senescence (Nakatogawa *et al.*, 2009; Reumann *et al.*, 2010) as well as for survival under some biotic/abiotic-stress conditions (Klionsky, 2004). Autophagy occurs by two main mechanisms, microautophagy and macroautophagy. In microautophagy, an invagination of the vacuolar membrane directly engulfs the cytosolic component to be degraded (Klionsky and Ohsumi, 1999). By contrast, in macroautophagy, autophagosomes form at the periphery of damaged or overproduced proteins. Autophagosomes enclose organelles or cytosolic compounds, which are transported into the vacuole and broken down by the non-selective degradation pathway (Meijer *et al.*, 2007). To date, more than 30 autophagy-associated (*atg*) genes have been identified in yeast and Arabidopsis (*Arabidopsis thaliana*) (Bassham *et al.*, 2006).

In pre-senescent leaves during vegetative growth, chloroplasts contain the majority of plant nutrients. For example, chloroplastic proteins contain 75–80% of total leaf nitrogen in C_3_ plants (Makino and Osmond, 1991). Thus, the degradation of chloroplast proteins in old or inefficient leaves during senescence provides important nutrients for relocation to developing organs. In recent years, the degradation mechanisms of chloroplasts and chloroplast proteins during senescence have been widely studied. During leaf senescence, Rubisco, the most abundant stromal protein in the chloroplasts (Wittenback, 1978), is released from chloroplasts into the cytoplasm as small double-membrane bodies termed Rubisco-containing bodies (RCBs; Chiba *et al.*, 2003) that are then transported to the central vacuole by autophagy for degradation (Ishida *et al.*, 2008). RCBs were not observed in the leaves of autophagy-defective *atg4* (Wada *et al.*, 2009) and *atg5* (Ishida *et al.*, 2008) mutants in *Arabidopsis*, indicating the direct involvement of macroautophagy in the degradation of Rubisco during leaf senescence. For the degradation of Rubisco and stromal proteins, another extra-chloroplastic degradation system, called senescence-associated vacuoles (SAVs), was also identified. SAVs are clearly smaller than the central vacuole and contain Rubisco and other stromal proteins, including glutamine synthetase, but not the photosystem proteins (Martinez *et al.*, 2008). This indicates that the SAV-dependent degradation system mainly functions in the degradation of stromal proteins in chloroplasts.

In contrast to the extra-chloroplastic degradation mechanisms for Rubisco and other proteins, an intra-chloroplastic degradation system mainly degrades thylakoid proteins. Plastids isolated from senescing leaves can degrade photosystem proteins under light conditions (Feller *et al.*, 2008) indicating that senescing chloroplasts have active systems to degrade photosystem proteins. This system may include the chloroplast protease FtsH, as the *Arabidopsis* T-DNA insertion KO mutants of *ftsH6* were unable to degrade Lhcb3 during dark-induced senescence and were also unable to degrade Lhcb1 and Lhcb3 under high light conditions (Zelisko *et al.*, 2005).

Chlorophyll (Chl) is degraded by several Chl catabolic enzymes (CCEs; Hörtensteiner, 2013). In addition, *STAY-GREEN* (*SGR*), Mendel’s green cotyledon gene encoding a novel chloroplast protein, functions in the initiation of Chl degradation (Park *et al.*, 2007; Ren *et al.*, 2007). Recently, it was demonstrated that SGR and six CCEs form a complex with light-harvesting complex II (LHCII), which may allow metabolic channelling of phototoxic Chl degradation intermediates (Sakuraba *et al.*, 2012b, 2013). Chl degradation ends with the formation of fluorescent chlorophyll catabolite (FCC), a non-toxic Chl degradation intermediate, in chloroplasts. For the final steps of Chl breakdown, FCC is transported into the vacuole and converted to non-fluorescent Chl catabolite (NCC) (Oberhuber *et al.*, 2003; Hörtensteiner and Krautler., 2011).

Although Chl breakdown generally occurs in the chloroplast until the formation of FCC, Wada *et al.* (2009) detected Chl fluorescence in the central vacuole of dark-induced senescing leaves in *Arabidopsis*, strongly indicating that macroautophagy also functions in the transport of Chl–apoprotein complexes from chloroplasts to the vacuole during senescence. Considering this function, the autophagy-dependent degradation system and other intra-chloroplastic degradation systems seem to share target proteins or macromolecules, including Chl and Chl-binding photosystem proteins. However, the relationship among these different degradation systems remains enigmatic.

It was found here that *atg5* mutants display a stay-green phenotype only under mild abiotic-stress conditions, but not under strong stress conditions; *atg5* leaves showed early leaf yellowing with extensive cell death under strong abiotic-stress conditions. Under mild abiotic-stress conditions, however, *atg5* acts as a functional stay-green mutant, maintaining the proper balance of Chls and photosynthetic proteins and retaining the grana thylakoid structure in the chloroplasts. Genetic analysis of *atg5* and the non-functional stay-green mutant *nye1-1* revealed that autophagy contributes to the non-selective breakdown of Chl-photosynthetic proteins during mild abiotic-stress-induced leaf yellowing, in addition to the selective breakdown of Chl-apoproteins through a dynamic STAY-GREEN1(SGR1)/NYE1-CCE complex in the senescing chloroplasts (Sakuraba *et al.*, 2012b). The relationship between autophagy-induced and SGR1-dependent degradation of the Chl-apoprotein complex in chloroplasts is also discussed.

## Materials and methods

### Plant materials and growth conditions

The *Arabidopsis thaliana* plants were grown on soil at 21–23 °C under long day (LD) conditions (16/8h light/dark; 90–100 μmol m^–2^ s^–1^ cool-fluorescent white light). For dark treatment, detached or attached leaves of 3-week-old plants were placed in complete darkness. Wild type, *atg5* and *nye1-1* are of the Col-0 ecotype. The *atg7* mutant (Ws-2 ecotype; CS39995) was obtained from the Arabidopsis Biological Resource Center (ABRC, Ohio, USA).

### Trypan blue staining

Leaves were incubated overnight in lactophenol-trypan blue solution (10ml lactic acid, 10ml glycerol, 10g phenol, and 10mg trypan blue dissolved in 10ml distilled water) (Koch and Slusarenko, 1990). Stained leaves were then boiled for 1min and then decolourized in 60% glycerol solution.

### Chlorophyll quantification

Chlorophylls (Chls) were extracted from leaf tissues with 80% ice-cold acetone solution at 4 °C. Chl concentration was quantified by a spectrophotometric method (Porra *et al.*, 1989).

### Immunoblot analysis

Protein extracts were prepared from rosette leaves of *Arabidopsis thaliana*. A 10mg aliquot of leaf tissue was ground in liquid nitrogen and homogenized with 100 μl of sample buffer [50mM TRIS–HCl, pH 6.8, 2mM EDTA, 10% (w/v) glycerol, 2% SDS, and 6% 2-mercaptoethanol] was used to suspend the protein extracts. The protein samples were subjected to SDS-PAGE. Gels were stained with Coomassie Brilliant Blue R-250 (Sigma–Aldrich). Antibodies against photosynthetic proteins, including Lhca3, Lhcb1, Lhcb2, Lhcb4, Lhcb5, CP43, D1, and PsaA (Agrisera, Sweden), were used for immunoblot analysis. Each protein was detected using an electrochemiluminescence (ECL) system (WESTSAVE, AbFRONTIER, Seoul, Korea) according to the manufacturer’s manual.

### Chl fluorescence measurement using pulse amplitude modulation

Maximal photochemical efficiency of PSII (*F*_v_/*F*_m_) was measured using the OS-30p+ instrument (OPTI-SCIENCES, USA). Detached leaves before and after salt treatment were adapted in the dark for 5min and the *F*_v_/*F*_m_ ratio was measured at room temperature. This 5min dark treatment resulted in the complete oxidation of *Q*_A_.

### Transmission electron microscopy

Transmission electron microscopy was conducted as previously described by Inada *et al.* (1998) with some modifications. Leaf tissues were fixed with modified Karnovsky’s fixative (2% paraformaldehyde, 2% glutaraldehyde, and 50mM sodium cacodylate buffer, pH 7.2). Samples were then washed with 0.05M sodium cacodylate buffer, pH 7.2, three times at 4 °C for 10min. The samples were post-fixed at 4 ºC for 2h with 1% osmium tetroxide in 0.05M sodium cacodylate buffer, pH 7.2, and washed twice with distilled water at room temperature. Samples were stained in 0.5% uranyl acetate at 4 °C overnight and dehydrated in an ethanol gradient and propylene oxide, then finally infiltrated with Spurr’s resin. Polymerization was performed at 70 °C for 24h and samples were sectioned with an ultramicrotome (MT-X). The sections were mounted on copper grids and stained with 2% uranyl acetate for 7min and with Reynolds’ lead citrate for 7min. Micrographs were made by using a LIBRA 120 transmission electron microscope (JEOL, Japan).

### Ion leakage measurement

To measure ion leakage after treatment, approximately 10 rosette leaves were placed in a tube with 6ml of 0.4mM mannitol. The tubes were placed at room temperature for 3h with shaking. Conductivity of the incubated solution was measured using an electroconductivity meter (CON6 METER, LaMOTTE Co., USA), before and after boiling for 10min.

### Abiotic-stress treatments

Analysis of salt stress was performed as previously described by Wu *et al.* (2012) with minor modifications. Detached leaves of 3-week-old plants were floated abaxial side-up, on 3mM MES buffer (pH 5.8) containing 150, 300, or 450mM NaCl. For osmotic stress, leaves were floated on buffer containing 50, 200, or 400mM mannitol. For oxidative stress, leaves were floated on buffer containing 5, 20, or 50mM H_2_O_2_.

### Reverse transcription (RT) and quantitative real-time PCR (qPCR) analysis

Total RNA was extracted from the leaf tissues using the Plant RNA Extraction Kit (iNtRON Biotechnology, Seoul, Korea) including the RNase-free DNase I treatment step to remove possible genomic DNA contamination. For RT, the first-strand cDNAs were prepared with 5 μg total RNA using M-MLV reverse transcriptase and aqn oligo(dT) primer (Promega). For quantitative real-time PCR (qPCR), 20 μl reactions, including first-strand cDNAs equivalent to 50ng total RNA, 10 μl 2× Universal SYBR Green Master Mix (Roche), and gene-specific forward and reverse primers (see Supplementary Table S1 at *JXB* online), were analysed using a Light Cycler 480 (Roche Diagnostics). Data analysis was conducted using the Roche Optical System software (ver. 1.5). The efficiency of qPCR analysis was calculated by comparing the slope of linear regression of C*t* and log_10_ of gene copies. Relative gene expression levels were normalized against the transcript levels of *GAPDH* (encoding glyceraldehyde phosphate dehydrogenase; At1g16300) as previously reported by Sakuraba *et al.* (2010).

## Results

### *atg5* leaves exhibit a stay-green phenotype under mild abiotic-stress conditions

Plant autophagy affects senescence and stress tolerance; *atg* mutants exhibit accelerated leaf yellowing during age- and dark-induced senescence (Thompson *et al.*, 2005), and hyper-sensitivity to abiotic stresses such as high salinity, oxidative stress, and drought (Xiong *et al.*, 2007; Liu *et al.*, 2009; Zhou *et al.*, 2013). This indicates that autophagy plays an important role in maintaining the proper balance of the cellular proteome during abiotic stresses. However, stress-induced chlorophyll (Chl) degradation should be impaired when autophagy does not operate properly, because autophagy is involved in chloroplast degradation, including Chl breakdown, during senescence (Ishida *et al.*, 2008; Wada *et al.*, 2009; Ono *et al.*, 2012).

**Fig. 1.**
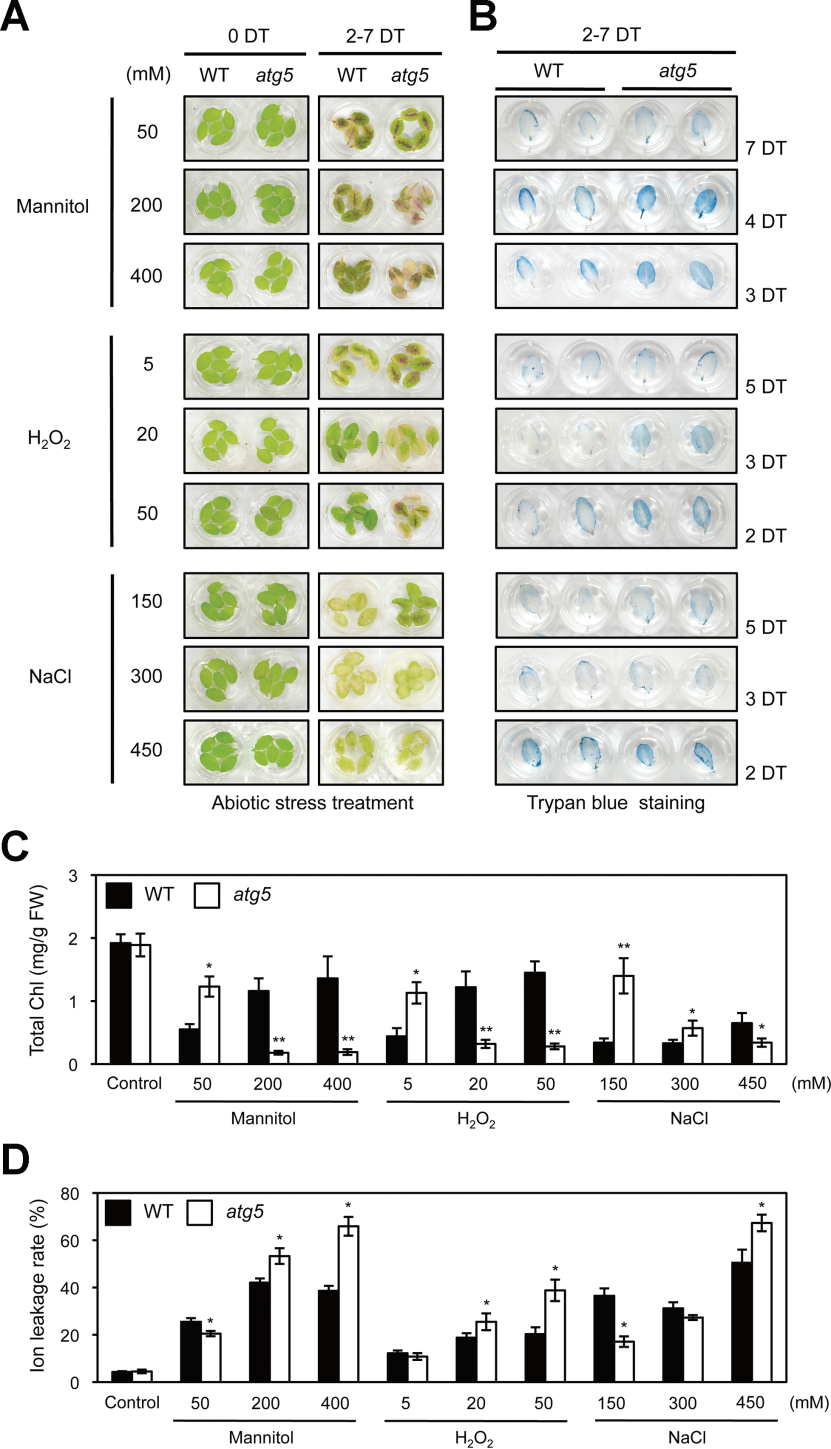
Phenotypic characterization of *atg5* leaves under different abiotic stresses. (A) Visible phenotypes of detached rosette leaves from 3-week-old wild-type (WT) and *atg5* mutants under osmotic (50, 200, or 400mM mannitol), oxidative (5, 20, or 50mM H_2_O_2_), and salt (150, 300, or 450mM NaCl) stress conditions. (B) Cell death in WT and *atg5* leaves under abiotic stresses, as shown by trypan blue staining. (C, D) The changes of total Chl levels (C) and ion leakage rates (D) in WT and *atg5* leaves after abiotic-stress treatments in (A). Black and white bars indicate WT and *atg5*, respectively. DT, days of treatment. Similar results were obtained from three independent experiments. Student’s *t*-test (*P<0.05, **P<0.01). (This figure is available in colour at *JXB* online.)

To investigate the relationship between autophagy and Chl degradation in more detail, we examined the visual phenotypes of *atg5* leaves under different abiotic-stress conditions (Fig. 1), including salt (NaCl), osmotic pressure (mannitol), and oxidative reagent (H_2_O_2_) treatments. To this end, the detached rosette leaves of 3-week-old plants were used to separate the effect of autophagy defects in each plant organ (Thompson *et al.*, 2005). As previously observed in whole plants (Thompson *et al.*, 2005; Zhou *et al.*, 2013), *atg5* leaves exhibited an early senescence phenotype under strong abiotic-stress conditions, such as 200 and 400mM mannitol, 20 and 50mM H_2_O_2_, or 450mM NaCl (Fig. 1A). Trypan blue staining revealed that early leaf yellowing of *atg5* leaves under these strong abiotic stresses resulted from cell death (Fig. 1B). By contrast, *atg5* leaves exhibited a stay-green phenotype under mild abiotic-stress conditions, such as 50mM mannitol, 5mM H_2_O_2_, or 150mM NaCl treatments (Fig. 1A). Notably, under these mild abiotic stresses, *atg5* leaves barely showed any cell death phenotype (Fig. 1B), suggesting that *atg5* is defective in Chl degradation, although only under mild abiotic-stress conditions in which accelerated cell death hardly occurs. To understand the stay-green phenotype of *atg5* leaves under mild stress conditions in more detail, total Chl levels and ion leakage rates were measured in each condition as an indicator of membrane disintegration and plant cell death. Consistent with the visible phenotypes, *atg5* leaves under mild abiotic-stress conditions had significantly higher total Chl levels than wild-type leaves (Fig. 1C). Compared with wild-type leaves, *atg5* leaves had significantly lower ion leakage rates, but had significantly higher rates under strong stress conditions (Fig. 1D). This confirms that the degree of cell death is closely associated with the phenotype of *atg5* leaves under these abiotic-stress conditions. *atg7*, another autophagy mutant, was also examined. Similar to *atg5* leaves, *atg7* leaves also showed a stay-green phenotype under mild abiotic-stress conditions (see Supplementary Fig. S1 at *JXB* online).

Our results using detached *atg5* leaves conflicted with previous results using whole plant bodies under salt-stress conditions (Zhou *et al.*, 2013). Therefore, the whole-plant phenotype of *atg5* mutants grown for 2 weeks on phytoagar plates containing a low concentration of NaCl (150mM) was examined. Consistent with the previous results (Zhou *et al.*, 2013), older leaves (cotyledon and 1st cycle of rosettes) of *atg5* plants showed a leaf necrosis phenotype (see Supplementary Fig. S2A and B at *JXB* online). However, younger leaves (2nd and 3rd cycle of rosettes) stayed green with higher Chl levels compared with those of wild-type leaves, suggesting that both detached and attached leaves of *atg5* have a stay-green capacity under mild salt-stress conditions, although *atg5* mutants exhibit a necrosis phenotype in older leaves.

### *atg5* exhibits a functional stay-green phenotype under mild abiotic-stress conditions

**Fig. 2.**
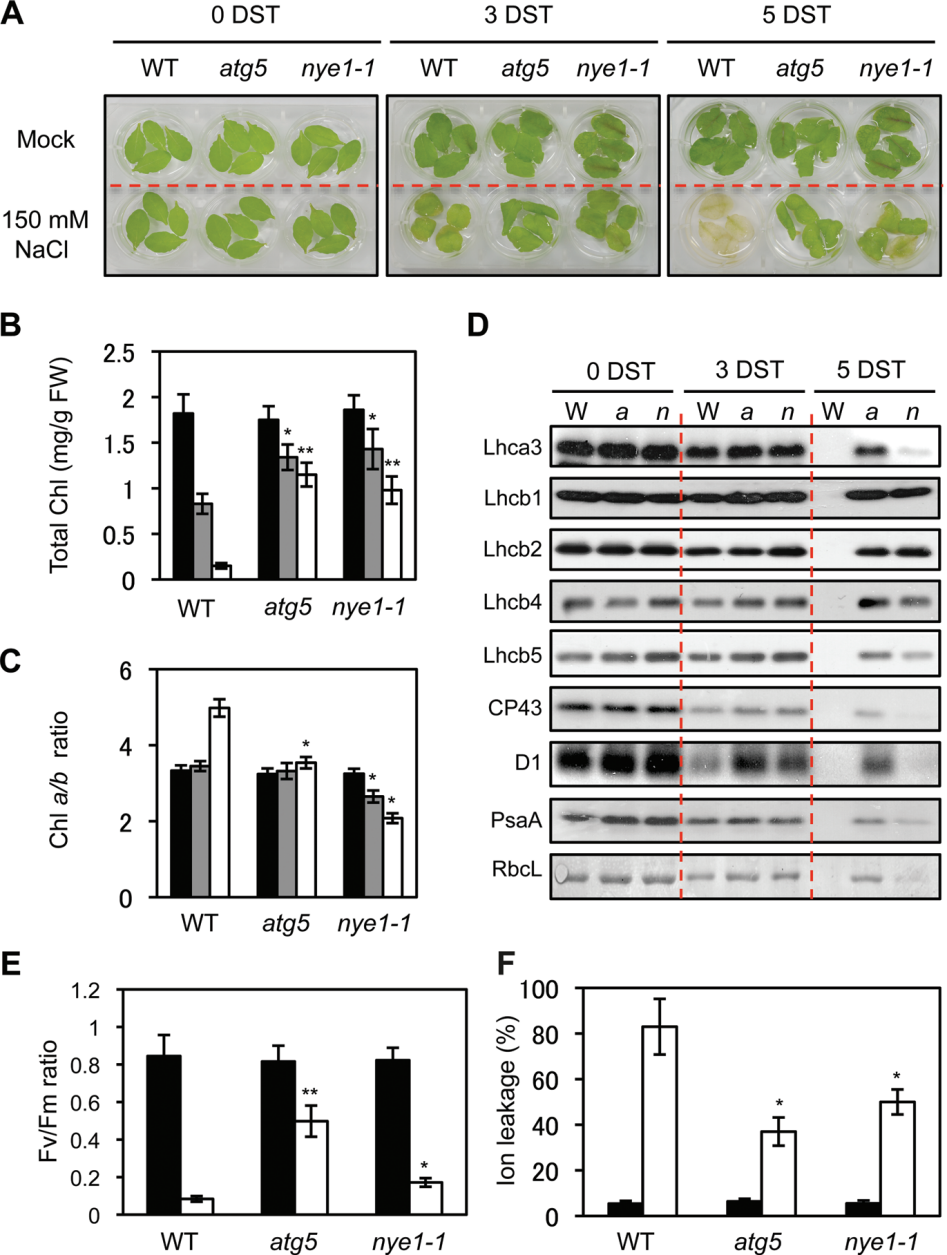
Characterization of *atg5* leaves under mild salt stress conditions. (A–F) Visible phenotypes (A), total Chl levels (B), Chl *a/b* ratios (C), photosynthetic protein levels (D), *F*_v_/*F*_m_ ratios (E), and ion leakage rates (F) of wild-type (WT), *atg5*, and *nye1-1* leaves under the mild salt-stress conditions. Detached leaves of 3-week-old WT, *atg5*, and *nye1-1* plants were incubated in 3mM MES buffer (pH 5.8) containing 150mM NaCl for 3 d and 5 d (3 and 5 DST, days of salt stress). (B, C, E, and F) Black, grey, and white bars indicate 0, 3, and 5 DST, respectively. (D) Antibodies against PSII core (CP43 and D1), PSII antenna (Lhcb1, Lhcb2, Lhcb4, and Lhcb5), PSI antenna (Lhca1 and Lhca3), and PSI core (PsaA) were used. RbcL (Rubisco large subunit) was visualized by Coomassie Brilliant Blue (CBB) staining after immunoblot analysis. These experiments were repeated more than three times with similar results. DST: days of salt treatment. Student’s *t*-test (*P<0.05, **P<0.01). (This figure is available in colour at *JXB* online.)

To characterize the stay-green phenotype of *atg5* leaves under mild abiotic stresses in more detail, several photosynthetic parameters of *atg5* were compared with a non-functional stay-green SGR1 mutant, *nye1-1* (Ren *et al.*, 2007), under mild salt-stress conditions (150mM NaCl). Similar to *atg5* mutants, *nye1-1* mutants also exhibited a stay-green phenotype after 3 d and 5 d of salt treatment (Fig. 2A). Consistent with the visible phenotype, *atg5* and *nye1-1* leaves showed significantly higher Chl retention than wild-type leaves (Fig. 2B).

**Fig. 3.**
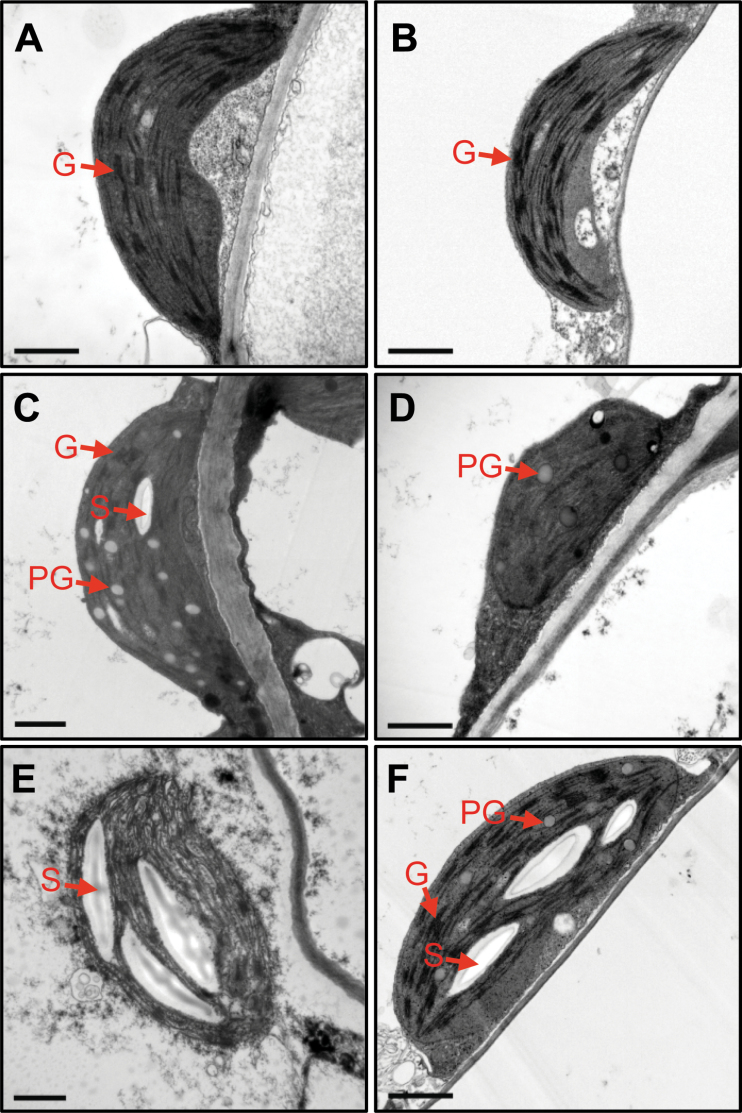
Transmission electron microscopy of plastids in *atg5* leaves under mild salt-stress conditions. (A, B) Chloroplasts in the mesophyll cells of 3-week-old wild-type (WT; A) and *atg5* (B) leaves before salt (150mM) treatment. (C–F) Chloroplasts in the mesophyll cells of WT (C, D, E) and *atg5* (F) leaves after 4 DST. DST, days of salt treatment; G, grana thylakoid; PG, plastoglobule; S, starch. Scale bars=1 μm. (This figure is available in colour at *JXB* online.)

Stay-green plants can be divided into functional and non-functional types (Thomas and Howarth, 2000; Hörtensteiner, 2009). The *Arabidopsis* SGR1 mutant, *nye1-1*, and the Chl catabolism-defective mutants, *nyc1-1* and *pph-1*, belong to the non-functional stay-green type (Kusaba *et al.*, 2007; Sato *et al.*, 2007; Morita *et al.*, 2009). Several photosynthetic parameters were therefore analysed to examine whether *atg5* is a functional or a non-functional stay-green type mutant under mild salt-stress conditions. First, the Chl *a/b* ratio of leaves was measured. Because SGR1/NYE1 contributes to Chl degradation in the light-harvesting complex of photosystem II (LHCII) with CCEs (Sakuraba *et al.*, 2012b), the Chl *a/b* ratio of *nye1-1* mutants gradually decreased under salt stress (Fig. 2C), and this selective stabilization of LHCII mainly contributes to a non-functional stay-green phenotype. By contrast, the Chl *a/b* ratio of *atg5* leaves did not change under salt stress. Similar to the Chl *a/b* ratio in *nye1-1*, LHCII proteins (Lhcb1, 2, 4, and 5) were predominantly retained while other photosystem proteins (CP43, D1, Lhca1, Lhca3, and PsaA) gradually decreased in *nye1-1* leaves during salt treatment (Fig. 2D). By contrast, *atg5* leaves retained all photosystem proteins at high levels (Fig. 2D), indicating that *ATG5* is involved in the non-selective destabilization of all photosystem proteins under salt stress-induced leaf senescence The Chl fluorescence parameter, *F*_v_ */F*_m_, representing the optimal yield of PSII, was then compared. After 4 d of salt treatment, the *F*_v_ */F*_m_ ratio in *atg5* leaves was higher than in wild-type and *nye1-1* leaves (Fig. 2E). The ion leakage rate, an indicator of membrane disintegration and one of the important factors for determining the stay-green type, was then examined. Ion leakage rates of *atg5* and *nye1-1* leaves were lower than for wild-type leaves (Fig. 2F). The chloroplast structure of *atg5* leaves was also examined. Before salt treatment, *atg5* leaves contained normal shapes of chloroplast and grana thylakoid structures, similar to wild-type leaves (Fig. 3A, B). After 4 d of salt treatment, grana thylakoids were hardly found in the chloroplasts of wild-type leaves, and different types of degrading chloroplasts were found (Fig. 3C–E). By contrast, *atg5* leaves retained the grana thylakoids (Fig. 3F).

Taken together, our results indicate that *atg5* acts as a functional stay-green type mutant under mild abiotic-stress conditions, because it retains a proper balance of photosystem proteins, photosynthetic efficiency and grana thylakoid structures.

### Expression of SAGs in *atg5* leaves under dark- or salt-stress-induced senescence conditions

**Fig. 4.**
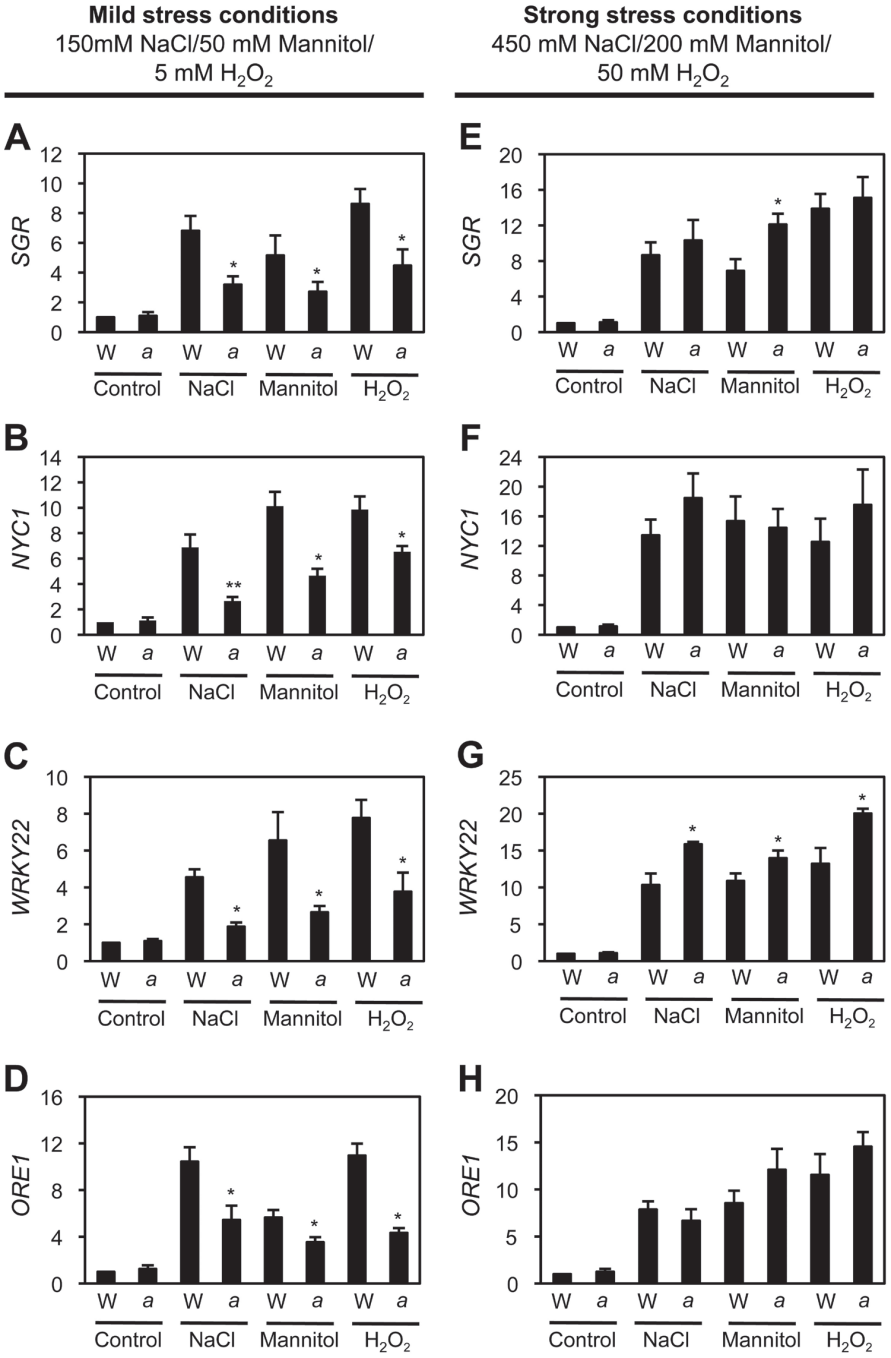
Altered expression of SAGs in *atg5* leaves under mild and strong abiotic-stress conditions. First-strand cDNAs were prepared from total RNA extracted from 3-week-old rosette leaves of WT and *atg5* plants before (control) and after 3 d of mild abiotic-stress treatments (A–D) and strong abiotic-stress treatments (E–H). By RT-qPCR analysis, relative expression levels of *SGR1/NYE1* (A, E), *NYC1* (B, F), *WRKY22* (C, G), and *ORE1* (D, H) were obtained by normalizing to the mRNA levels of *GAPDH*. Mean and SD values were obtained from more than three biological replicates. These experiments were replicated at least twice with similar results. Student’s *t*-test (*P<0.05, **P<0.01).

To reveal the mechanism of conditional delayed senescence in *atg5* leaves under mild abiotic-stress conditions, expression levels of senescence-associated genes (SAGs), including two senescence-induced TFs, *WRKY22* (Zhou *et al.*, 2011) and *ORE1* (Kim *et al.*, 2009), and two Chl catabolism-associated proteins, *SGR1/NYE1* (Park *et al.*, 2007; Ren *et al.*, 2007) and *NYC1* (Kusaba *et al.*, 2007), were measured under mild stress-induced senescence conditions. Expression levels of all four SAGs in wild-type leaves drastically increased after 3 d of mild stress conditions, such as 150mM NaCl, 50mM mannitol, and 5mM H_2_O_2_ treatments. However, their expression levels were significantly down-regulated in *atg5* leaves (Fig. 4A–D), indicating that the down-regulation of SAGs contributes to the functional stay-green phenotype of *atg5* leaves under mild abiotic-stress conditions. Although the non-functional stay-green *nye1-1* mutant also retained leaf greenness (Fig. 4), it was found that, under mild abiotic stresses, expression levels of *WRKY22* and *ORE1* in *nye1-1* leaves were almost the same as those of wild-type leaves (see Supplementary Fig. S3 at *JXB* online). The expression levels of four SAGs were also checked under strong abiotic-stress conditions. Except for the slight up-regulation of *WRKY22*, the expression levels of the other three SAGs were not significantly different in *atg5* leaves and wild-type leaves (Fig. 4E–H).

The stay-green phenotype in *atg5* leaves under mild stress conditions could also be caused by defects of other intra-chloroplastic catabolic systems. The expression levels of genes encoding FtsH proteases (*FtsH2* and *FtsH6*), Clp proteases (*ClpP4* and *ClpC1*), and Deg proteases (*DegP4* and *DegP8*) were also examined. Expression levels of these genes significantly increase under high light, cold, and heat-stress conditions (Sinvany-Villalobo *et al.*, 2004), indicating that these chloroplastic proteases have major roles in protein degradation under several abiotic-stress conditions. Under mild salt stress (150mM NaCl), the gene expression levels in *atg5* leaves were almost the same as those in wild-type leaves (see Supplementary Fig. S4 at *JXB* online), indicating that these three catabolic systems are not related to the functional stay-green phenotype of *atg5* leaves under mild abiotic-stress conditions.

Taking these results together, it is possible that the down-regulation of several SAGs in *atg5* leaves during mild abiotic-stress conditions is controlled by retrograde signalling between chloroplasts and the nucleus, which is often observed in many functional stay-green mutants (Sakuraba *et al.*, 2012a).

### Early leaf yellowing of *atg5* is independent of Chl breakdown in LHCII by the SGR1–CCEs complex in chloroplasts

Chl degradation and autophagy (macroautophagy) affect the degradation of Chls and photosynthetic proteins in the chloroplasts, a very late step in leaf senescence (Lim *et al.*, 2007). However, *atg5* and *nye1-1* retain different levels of photosynthetic proteins under mild salt-stress conditions (Fig. 2). Among photosynthetic proteins, *nye1-1* selectively retains LHCI and LHCII under salt stress (Fig. 2E), as the *nye1-1* mutant has impaired SGR1 function. SGR1 induces Chl degradation in LHCII by recruiting Chl catabolic enzymes (CCEs) (Sakuraba *et al.*, 2012b, 2013). However, most photosynthetic proteins are retained in *atg5* leaves with a constant Chl *a/b* ratio (Fig. 2), indicating that the degradation of photosynthetic proteins by the SGR1–CCE complex and macroautophagy may function independently.

**Fig. 5.**
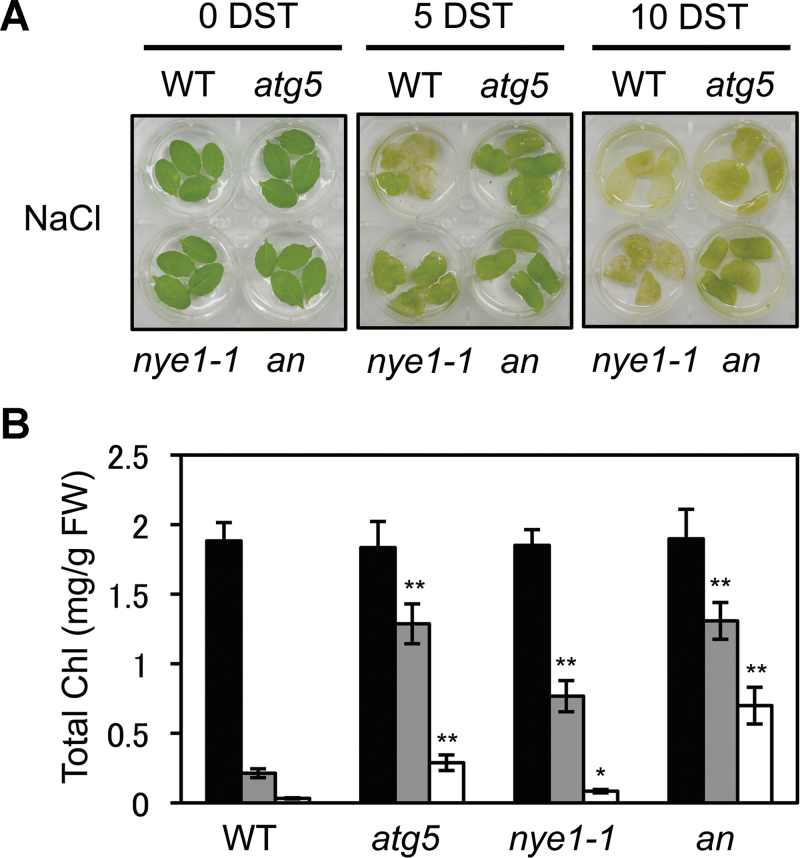
Characterization of *atg5 nye1-1* double mutant under mild salt-stress conditions. (A, B) Visible phenotypes (A) and total Chl levels (B) of detached leaves from wild-type (WT), *atg5*, *nye1-1*, and *atg5 nye1-1* (*an*) plants during the mild salt stress. Detached leaves from 3-week-old plants were incubated abaxial side-up on 3mM MES (pH 5.8) buffer containing 150mM NaCl for 5 d and 10 d. Similar results were obtained from three independent experiments. DST, days of salt treatment. Student’s *t*-test (*P<0.05, **P<0.01). (This figure is available in colour at *JXB* online.)

To examine this possibility, the phenotype of *atg5 nye1-1* double mutants was investigated under mild salt stress (150mM NaCl). Although no differences were observed between *atg5* single and *atg5 nye1-1* double mutants until 5 d of salt treatment, the double mutant exhibited a stronger stay-green phenotype at 10 d (Fig. 5A) with significantly greater retention of Chls (Fig. 5B), indicating that the two mutations show an additive effect on abiotic-stress-induced leaf senescence. To examine the relationship of the two mutations in more detail, the phenotype of the double mutants was checked under dark-induced senescence conditions. During dark-induced senescence, the *nye1-1* mutant shows a stay-green phenotype (Ren *et al.*, 2007), but *atg5* shows an early leaf yellowing phenotype (Thompson *et al.*, 2005). It was found that, after 4 d of dark incubation, the double mutant exhibited an intermediate phenotype and Chl levels of the two single mutants (see Supplementary Fig. S5 at *JXB* online). Collectively, these results indicate that Chl degradation during senescence occurs by two independent processes, SGR1–CCE–LHCII interaction and macroautophagy.

## Discussion

### *atg5* acts as a functional stay-green mutant under mild abiotic-stress conditions

Defects in chloroplast destruction or senescence-promoting mechanisms can cause leaves to retain their green colour during senescence, a phenomenon called ‘stay-green’ (Hörtensteiner, 2009). For example, the knockout mutants of CCEs exhibit a stay-green phenotype during dark-induced and natural senescence because of the impairment of Chl degradation (Schelbert *et al.*, 2009; Horie *et al.*, 2009). Because the degradation of chloroplast components involves autophagy (Ishida *et al.*, 2008; Wada *et al.*, 2009), it was expected that *atg* mutants would show a stay-green phenotype under senescence-inducing conditions. However, under strong stress conditions, the *atg* mutants exhibit a phenotype of accelerated yellowing and cell death.

**Fig. 6.**
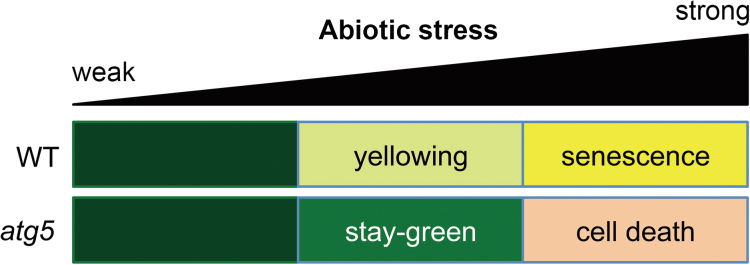
Leaf phenotypes of *atg5* mutants depending on the strength of abiotic stresses. Under strong abiotic-stress conditions, *atg5* leaves show an accelerated cell death phenotype; under mild abiotic-stress conditions, *atg5* leaves show little or no cell death, which leads to a stay-green phenotype. (This figure is available in colour at *JXB* online.)

In this study, this apparent inconsistency has been addressed by identifying the conditions under which *atg* mutants show a stay-green phenotype. Under mild abiotic-stress conditions, such as 150mM NaCl, 5mM H_2_O_2_, or 50mM mannitol, *atg5* leaves exhibit a stay-green phenotype (Fig. 1A), and show little cell death (Fig. 1B). Under strong abiotic-stress conditions, however, *atg5* leaves turn yellow much faster than WT leaves and show extensive cell death (Fig. 1). This rapid leaf yellowing phenotype is consistent with previous studies on the phenotype of *atg* plants under abiotic-stress conditions (Xiong *et al.*, 2007; Liu *et al.*, 2009; Zhou *et al.*, 2013). It is speculated that the difference in *atg* phenotype between strong- and weak-stress conditions may reflect the importance of autophagy in adapting to severe-stress conditions. Generally, plants need to change the balance of the proteome drastically under abiotic-stress conditions, for adaptation to these extreme environments. Because *atg* mutants cannot properly control their proteome balance, they cannot adapt to strong abiotic-stress conditions and thus exhibit extensive cell death. However, adaptation to mild-stress conditions does not require drastic changes in the proteome balance. Thus, *atg5* leaves exhibit little cell death, which leads to the stay-green phenotype (Fig. 6).

Autophagy is involved in chloroplast degradation, a downstream step in leaf senescence pathways. It was therefore expected that the stay-green phenotype of *atg* mutants would resemble the phenotypes of the cosmetic stay-green mutants of SGR1 and CCEs (Kusaba *et al.*, 2007; Morita *et al.*, 2009). However, it was found that *atg5* conditionally acts as a functional, not a cosmetic stay-green type mutant. Under mild salt stress (150mM NaCl) conditions, *atg5* leaves stayed green with the proper balance of photosynthetic proteins (Fig. 2D), high photosynthetic capacity (Fig. 2E), and well-retained grana thylakoids (Fig. 3). Also, *atg5* leaves showed lower expression levels of several SAGs under mild abiotic-stress conditions (Fig. 4), indicating that *atg5* leaves during mild abiotic-stress conditions show defects in both autophagy-dependent senescence pathways and other senescence pathways. One possibility is that the retained chloroplast proteins in *atg5* leaves may induce retrograde signalling from the chloroplasts to nucleus, leading to the altered expression of the SAGs. Recently, chloroplast homeostasis has been implicated as an important factor in leaf senescence; for example, tobacco plants with reduced NADH dehydrogenase activity exhibited delayed senescence without significant alteration of their growth rate (Zapata *et al.*, 2005). In addition, *Arabidopsis* plants over-expressing chlorophyllide *a* oxygenase (CAO) showed changes in the Chl pigment composition of the photosynthetic apparatus and also showed a functional stay-green phenotype with wide changes in SAG expression (Sakuraba *et al.*, 2012a). These results indicate that chloroplasts have an important role in regulating nuclear gene expression during leaf senescence.

Together, these data indicate that the multiple effects of two different pathways probably cause the functional stay-green phenotype of *atg5* leaves under mild abiotic-stress conditions. Because the macroautophagy pathway does not function in *atg5* leaves, the degradation of chloroplasts and chloroplastic proteins itself is impaired. Simultaneously, the retention of chloroplast proteins induces chloroplast–nucleus retrograde signalling that affects the regulation of SAGs. Some enigmas remain for this hypothesis; for instance, the chloroplast component(s) that mediate the chloroplast–nucleus retrograde signalling during leaf senescence remain to be identified. Further physiological and biochemical analyses of the functional stay-green phenotype of *atg* mutants are essential for revealing the functions of plant autophagy.

### Degradation of Chls and photosynthetic proteins requires both autophagy and intra-chloroplastic catabolic systems during leaf senescence

SGR1/NYE1 and CCEs form a dynamic protein complex for LHCII disassembly and Chl degradation (Sakuraba *et al.*, 2012a, 2013). However, the SGR1–CCE complex does not interact with other photosynthetic proteins, such as LHCI, the PSII core complex, or the PSI core complex (Sakuraba *et al.*, 2012a). Consistent with this LHCII-specific interaction, LHCII proteins were dominantly retained while other photosystem proteins were normally degraded in *nye1-1* (Fig. 2D) and a CCE mutant *nyc1-1* (Horie *et al.*, 2009). Thus, degradation mechanisms of other photosynthetic proteins and Chls still remain enigmatic.

At least in part, our finding of the functional stay-green phenotype in *atg5* leaves under mild salt-stress condition provides an important clue to solve this enigma. The levels of several photosynthetic proteins retained in *atg5* and *nye1-1* leaves under mild salt stress were compared. Although both *atg5* and *nye1-1* leaves exhibited a stay-green phenotype (Fig. 2A) with highly retained Chl levels (Fig. 2B), only LHCII proteins were predominantly retained in *nye1-1* leaves, whereas all photosynthetic proteins were substantially retained in *atg5* leaves (Fig. 2D). Recent reports showed that one of the autophagy pathways, the so-called chlorophagy pathway, functions in the transportation of Chls and photosystem proteins from the chloroplasts to the vacuole for their degradation (Wada *et al.*, 2009). Considering the low selectivity of proteolysis in autophagy, the chlorophagy pathway non-selectively transported all photosystem proteins from the chloroplasts to the vacuole. Although RCBs also act in chloroplastic autophagy, RCBs do not show Chl fluorescence (Ishida *et al.*, 2008). SAVs, another extra-chloroplastic catabolic system, contain Chl *a*, but not photosystem proteins. Thus, so far, chlorophagy is the only identified extra-chloroplastic degradation system for photosystem proteins. By contrast with chlorophagy, the SGR–CCE complex seems to concentrate on the destabilization of LHCII and Chls. LHCII, especially the three major, abundant subunits (Lhcb1, Lhcb2, and Lhcb3), forms aggregates because of its abundance (Ruban *et al.*, 2012). In this sense, if specific degradation systems for LHCII exist, it is natural that the SGR1–CCE complex would be one of them.

In this study, it was also found that *atg5 nye1-1* double mutants exhibited a very strong stay-green phenotype under mild salt-stress conditions, a seemingly additive phenotype (Fig. 5). This result indicates that the autophagy- and SGR1-dependent degradation pathways function independently. Reflecting the relationship of these two pathways, the combination of intra- and extra-chloroplastic catabolic pathways acts to degrade photosystem proteins. Another catabolic system may also function in the degradation of photosystem proteins during leaf senescence. For instance, genes encoding several members of the FtsH, Clp, and Deg protease families were significantly up-regulated in senescing leaves (Gepstein *et al.*, 2003; Andersson *et al.*, 2004) strongly indicating that these chloroplastic proteases have important roles in the degradation of chloroplast proteins during leaf senescence as well as abiotic-stress conditions. Indeed, an FtsH protease affects the turnover of D1 protein, one of the core subunits of photosystem II, under abiotic-stress conditions (Bailey *et al.*, 2002; Kato *et al.*, 2009). Thus, it is possible that these proteases are involved in the degradation of photosystem proteins during senescence.

Further biochemical analyses of chloroplasts and vacuole fractions during senescence will help us to understand the complete picture of the degradation mechanisms of photosynthetic proteins and their photosynthetic pigments.

## Supplementary data

Supplementary data can be found at *JXB* online.

Supplementary Fig. S1. Phenotype of wild-type (WT) and *atg7* leaves under mild abiotic-stress conditions.

Supplementary Fig. S2. Phenotype of wild-type (WT) and *atg5* plants grown on phytoagar plates containing NaCl.

Supplementary Fig. S3. Altered expression of SAGs in *nye1-1* leaves under mild abiotic-stress conditions.

Supplementary Fig. S4. Expression analysis of chloroplastic protease genes in *atg5* leaves under mild salt -stress conditions.

Supplementary Fig. S5. Phenotype (A) and total Chl level (B) of *atg5 nye1-1* double mutants during dark-induced senescence.

Supplementary Table S1. Primers used for qPCR in this study.

Supplementary Data
