# Supplementary material for: Delayed degradation of chlorophylls and photosynthetic proteins in Arabidopsis autophagy mutants during stress-induced leaf yellowing
Source: J Exp Bot. 2014 Feb 8;65(14):3915–25. doi: 10.1093/jxb/eru008 (PMC4106435; doi:10.1093/jxb/eru008)
Supplement: Supplementary Data [file supp_65_14_3915__index.html]

Delayed degradation of chlorophylls and photosynthetic proteins in Arabidopsis autophagy mutants during stress-induced leaf yellowing — Delayed degradation of chlorophylls and photosynthetic proteins in Arabidopsis autophagy mutants during stress-induced leaf yellowing — Supplementary Data 

# Delayed degradation of chlorophylls and photosynthetic proteins in *Arabidopsis* autophagy mutants during stress-induced leaf yellowing

## Supplementary Data

Data files

**Files in this Data Supplement:**

- Supplementary Data - Supplementary Data
