# Supplementary material for: Delayed degradation of chlorophylls and photosynthetic proteins in Arabidopsis autophagy mutants during stress-induced leaf yellowing
Source: J Exp Bot. 2014 Feb 8;65(14):3915–25. doi: 10.1093/jxb/eru008 (PMC4106435; doi:10.1093/jxb/eru008)
Supplement: Supplementary Data [file supp_eru008_jexbot111633_file001.pdf]

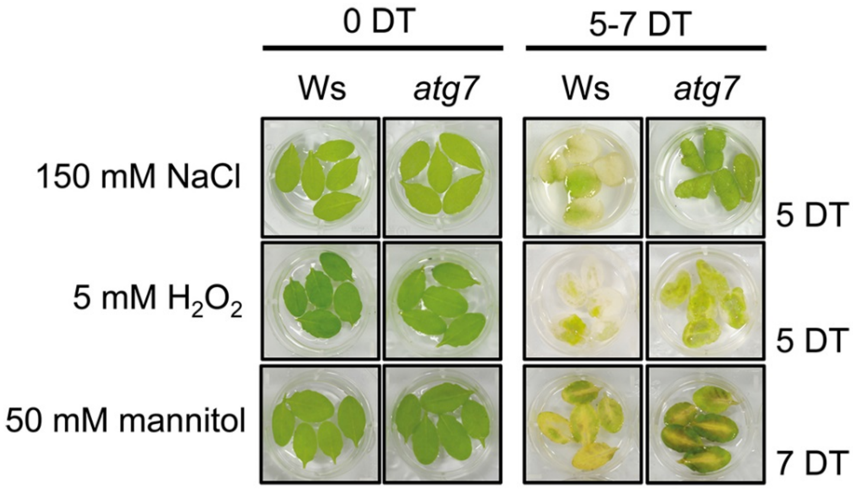

Supplementary Fig. 1. Phenotype of wild-type (WT) and *atg7* leaves under mild abiotic stress conditions.

Detached leaves of 3-week-old WT and *atg7* plants were incubated abaxial side-up on 3 mM MES (pH 5.8) buffer with 50 mM mannitol, 5 mM H_2O_2, or 150 mM NaCl, respectively. *atg7* leaves show a stay-green phenotype under mild abiotic stress conditions, very similar to *atg5* leaves. DT, days of treatment.

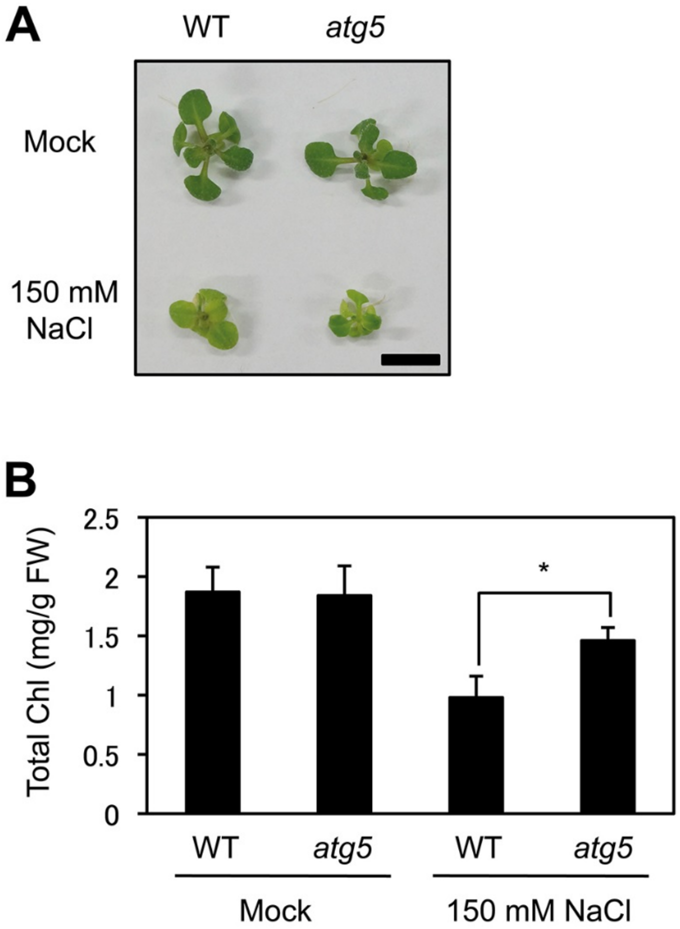

Supplementary Fig. 2. Phenotype of wild-type (WT) and *atg5* plants grown on phytoagar plates containing NaCl.

Phenotypes (A) and total Chl levels (B) of 2-week-old WT and *atg5* plants grown on agar plates with or without 100 mM NaCl under LD conditions. (A) Black bars = 1cm. (B) For Chl quantification, 2nd cycle of rosettes were used. Student's *t*-test (*P < 0.05).

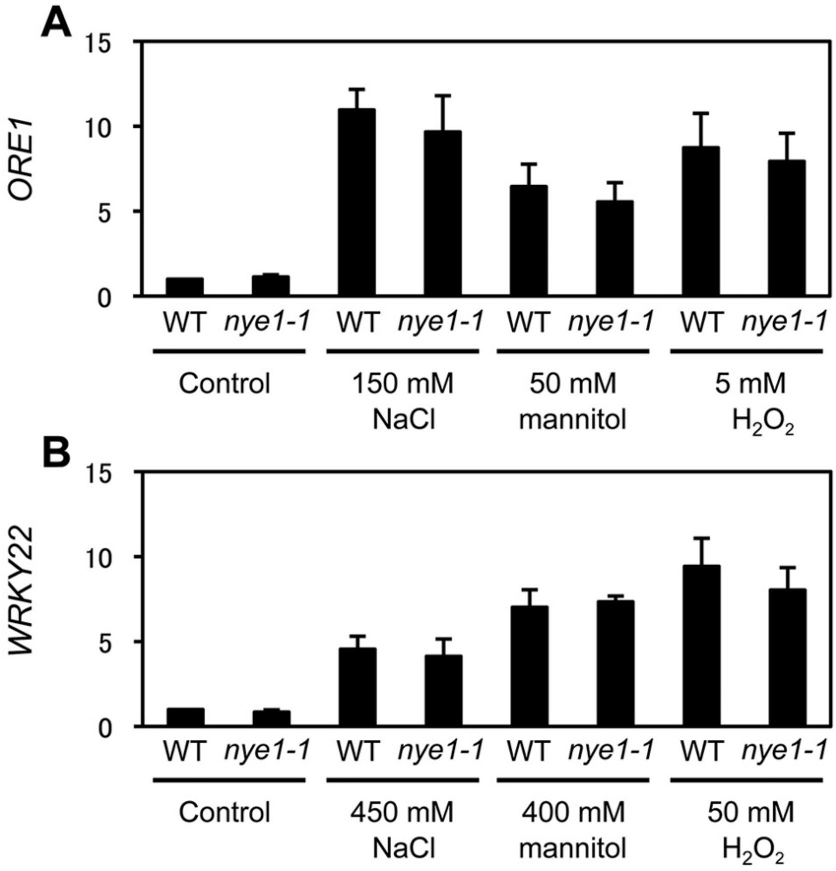

Supplementary Fig. 3. Altered expression of SAGs in *nye1-1* leaves under mild abiotic stress conditions.

First-strand cDNAs were prepared from total RNA extracted from 3-week-old rosette leaves of WT and *nye1-1* plants before and after 3 days of mild abiotic stress treatment (150 mM NaCl, 50 mM mannitol, and 5 mM H_2O_2). After RT-qPCR, relative expression levels of *ORE1* (A) and *WRKY22* (B) were obtained by normalizing to the transcript levels of *GAPDH*. Means and SDs were obtained from more than three biological replicates. These experiments were replicated at least twice with similar results.

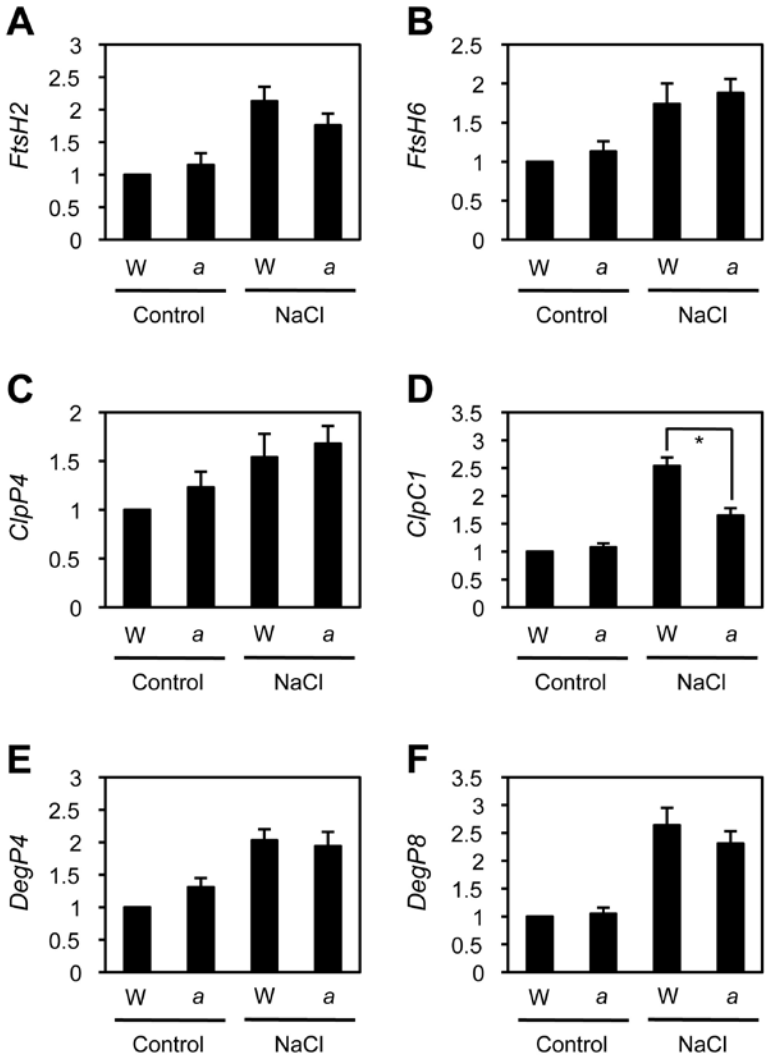

Supplementary Fig. 4. Expression analysis of chloroplastic protease genes in *atg5* leaves under mild salt stress conditions.

First-strand cDNAs were prepared with total RNA extracted from 3-week-old rosette leaves of WT and *atg5* leaves before and after 3 days of mild salt stress (150 mM NaCl). After RT-qPCR, relative expression levels of *FtsH2* (A), *FtsH6* (B), *ClpP4* (C), *ClpC1* (D), *DegP4* (E), and *DegP8* (F) were obtained by normalizing to the transcript levels of *GAPDH*. Means and SDs were obtained from more than three biological replicates. These experiments were replicated at least twice with similar results. Student's *t*-test (*P < 0.05).

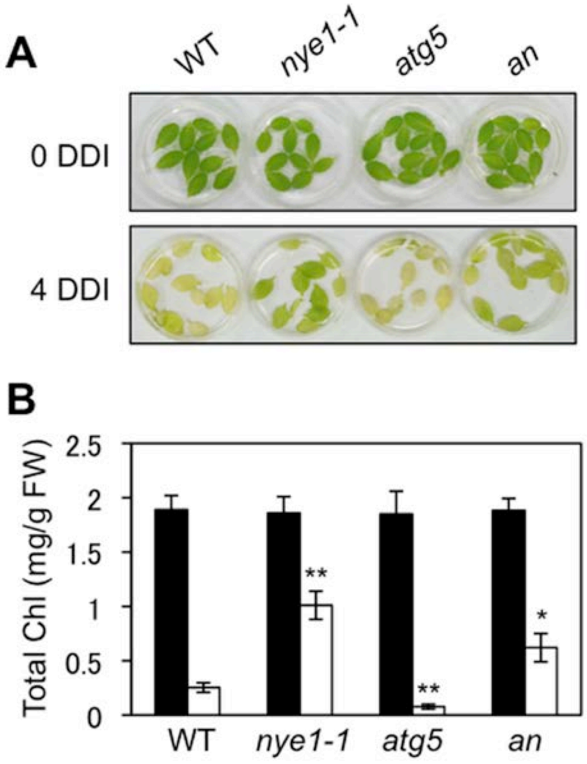

Supplementary Fig. 5. Phenotype (A) and total Chl level (B) of *atg5 nye1-1* double mutants during dark-induced senescence.

Detached leaves from 3 week-old WT, *atg*5, *nye1-1*, and *atg5 nye1-1* plants were incubated abaxial side-up on 3 mM MES (pH 5.8) buffer under dark conditions (dark-induced senescence, Figure S4A) for 4 days. DDI, Days of dark incubation. Student's *t*-test (*P < 0.05, **P < 0.01).

Supplementary Table 1. Primers used for qPCR in this study.

Gene	Forward primer (5' → 3’)	Reverse primer (5’→3’)
ATG5	TCTCAACAAGTTGTGCCTGAG	GTACGAGATGTCATCCCAGGT
SGR1	TGGGCAAATAGGCTATACCG	CCACCGCTTATGTGACAATG
NYC1	GTTAACAGACGCGATGGAGA	GCCTGGAAAAGAGCTAGGTG
ORE1	AATGAAGCTGTTGCTTGACG	AGAAATTCCAAACGCAATCC
WRKY22	TACGGACAGAAACCCATCAA	CATCTTCGGGTCGGATCTAT
FtsH2	AGAAACTATTGGCGGTGACG	TGATGCTGGAGTTGTCGTTG
FtsH6	AGCAAGACAGATGGTGACGA	GCAAGTTTCTCGGACATTGA
ClpP4	ATGCCCAACACGAGGATAAT	AGTACATCCCGCGATAATGC
ClpC1	GCCACTTCCACCATTTAGCA	TAACCCGAGCTATGGAGCAA
DegP4	TTCTCGCAATTGATGATGTTC	TGCTCTTTCCCATCTCTCAA
DegP8	TTCGTAATGGAGCCCTTGTC	CGGCTTTGTTCTTCACAGGT
